# Association between duration of breastfeeding and malocclusions in primary and mixed dentition: a systematic review and meta-analysis

**DOI:** 10.1038/s41598-017-05393-y

**Published:** 2017-07-11

**Authors:** Montserrat Boronat-Catalá, José María Montiel-Company, Carlos Bellot-Arcís, José Manuel Almerich-Silla, Montserrat Catalá-Pizarro

**Affiliations:** Department of Stomatology, Faculty of Medicine and Dentistry, University of Valencia, Valencia, Spain

## Abstract

The aim of this systematic review and meta-analysis was to examine the current evidence on the possible effects of breastfeeding on different malocclusion traits in primary and mixed dentition. A systematic search was made in three databases, using terms related to breastfeeding and malocclusion in primary and mixed dentition. Of the 31 articles that met the inclusion criteria and were included in the qualitative analysis, nine were included in the quantitative analysis. The quality of the 31 observational studies was moderate to high on the Newcastle-Ottawa Scale. It was found that the odds ratio for the risk of posterior crossbite was 3.76 (95% CI 2.01–7.03) on comparing children who had not been breastfed, with those breastfed for over six months, and rose to 8.78 (95% CI 1.67–46.1) when those not breastfed were compared to those breastfed for over twelve months. The odds ratio for class II malocclusion in children breastfed for up to six months compared to those breastfed for over six months was 1.25 (95% CI 1.01–1.55). Lastly, children who were breastfed for up to six months had an odds ratio of 1.73 (95% CI 1.35–2.22) for non-spaced dentition compared to those who were breastfed for over six months.

## Introduction

The WHO recommends exclusive breastfeeding for at least the first six months of life, as this reduces the risk of infectious diseases of the gastrointestinal tract and respiratory system^1^.

Craniofacial development involves functional stimuli such as respiration, mastication, sucking and swallowing^2^. In addition to the protection against infection afforded by breastfeeding, it has also been observed to promote correct craniofacial development owing to the intense muscular activity it requires, which favors proper lip closure, stimulates mandibular function and positions the tongue correctly against the palate^3^. The movements of lips and tongue during breastfeeding mean that the child obtains milk through a “squeeze action”, whereas bottle-fed children make a more passive movement to obtain the milk, causing less stimulation of the orofacial structures^4^.

Consequently, breastfeeding can promote better occlusal development and correct growth of the orofacial structures, and the better occlusal development can extend through into the mixed dentition stage. Nevertheless, the influence of breastfeeding on occlusion is a subject of debate in the scientific literature.

Some authors have studied the relationship between breastfeeding and occlusion and reached very different conclusions, from the absence of any association between breastfeeding and occlusion^5–7^ to a specific relationship between a shorter duration of breastfeeding and the appearance of particular types of malocclusion, such as posterior crossbite^8–11^, open bite^12^ or class II malocclusion^13, 14^.

Owing to this controversy, the aim of the present systematic review and meta-analysis is to examine the current evidence on the possible effects of breastfeeding on the different malocclusion traits in primary and mixed dentition.

## Methods

A systematic review of the literature was conducted in accordance with the MOOSE guidelines for Meta-analyses and Systematic Reviews of Observational Studies^15^. The review was registered with the PRISMA (PROSPERO) database under number CRD42016032862.

### Review questions

A PICO question was formulated as follows: Population – children with primary or mixed dentition; Exposure – duration of breastfeeding; Comparison – absence of breastfeeding; Outcome – malocclusions. Is breastfeeding a protective factor against malocclusion in primary and mixed dentition? Does the duration of breastfeeding have an effect on occlusal development? What occlusal traits of primary and mixed dentition are influenced by breastfeeding?

### Criteria for inclusion and exclusion

The inclusion criteria were: randomized controlled trials (RCTs), case-control studies and cohort studies in humans. Retrospective and prospective studies were both included. Systematic reviews, meta-analyses, clinical cases, case series, literature reviews and editorials were excluded. Studies that assessed the current evidence on the effect of breastfeeding on occlusion in primary and mixed dentition were included. Both exclusive and mixed breastfeeding were included in order to assess the effects on occlusion in relation to the number of months of breastfeeding.

### Search strategy

To identify relevant studies irrespective of language, a rigorous electronic search was made in the Pubmed, Embase and Scopus databases. An electronic search for “grey literature” was also made in the New York Academy of Medicine Grey Literature Report. The search was made with no time limit on January 30, 2017. The following search strategy was employed: (child* OR infant OR infant, newborn OR baby) AND (breastfeed* OR breast feeding) AND (dental occlusion OR occlus* OR malocclusion OR crossbite* OR bite, cross OR bites, cross OR overbite OR deep bite OR dental overjet OR incisor protrusion OR open bite). For this search strategy, both MeSH and non-MeSH terms were used. Attention was paid to the different combinations of these terms and the electronic search was complemented by hand searching for the bibliographical references cited in the eligible articles in order to add studies that had not been found during the primary search.

Two independent reviewers (MBC and MCP) assessed the titles and abstracts of all the articles selected. The Kappa score^16^ was used to assess the degree of agreement on eligibility on reading the title and abstract. In the event of disagreement, a third reviewer (CBA) was consulted. If the information provided by the abstract was insufficient to reach a conclusion, the reviewers read the full article before taking the final decision. Subsequently, the full texts of all the articles were read and the reasons for rejecting those excluded were recorded.

### Data extraction and list of variables

The following data were extracted from each of the studies included: author, year of publication, type of study, type of dentition studied (primary or mixed), sample size, sample selection method, gender, mean age, dropouts, type of malocclusion studied, breastfeeding data collection method, results, breastfeeding-malocclusion odds ratio (OR) and quality of the article.

### Quality assessment

The quality of the studies was evaluated on the Newcastle-Ottawa Quality Assessment Scale (NOS) for cohort and case-control studies^17^. The NOS contains eight items. Each study can be awarded only one star for each item, with the exception of Comparability, for which up to two stars can be given, so the maximum possible score for each study is nine stars. The quality of the studies was assessed independently by two reviewers. If they disagreed, a consensus was reached with a third reviewer.

### Measurement of the variables and synthesis of the results

The odds ratios of associations between different lengths of breastfeeding (no breastfeeding, less than six months’ breastfeeding, over six months’ breastfeeding, over twelve months’ breastfeeding) and class II molar, openbite, non-spaced dentition and posterior crossbite were calculated.

### Statistical analysis

The Odds Ratios were estimated using the calculadora_metaanalisis.xls calculator from CASPe (Critical Appraisal Skills Programme Español). Heterogeneity was assessed with the Q test, at p < 0.1, as well as the I^2^ test. For the combination of studies, the DerSimonian-Laird random effects pooling method was used to calculate the weighted odds ratio. Rosenthal’s tolerance level was employed to assess publication bias.

## Results

### Study selection and flow diagram

**Figure 1:**
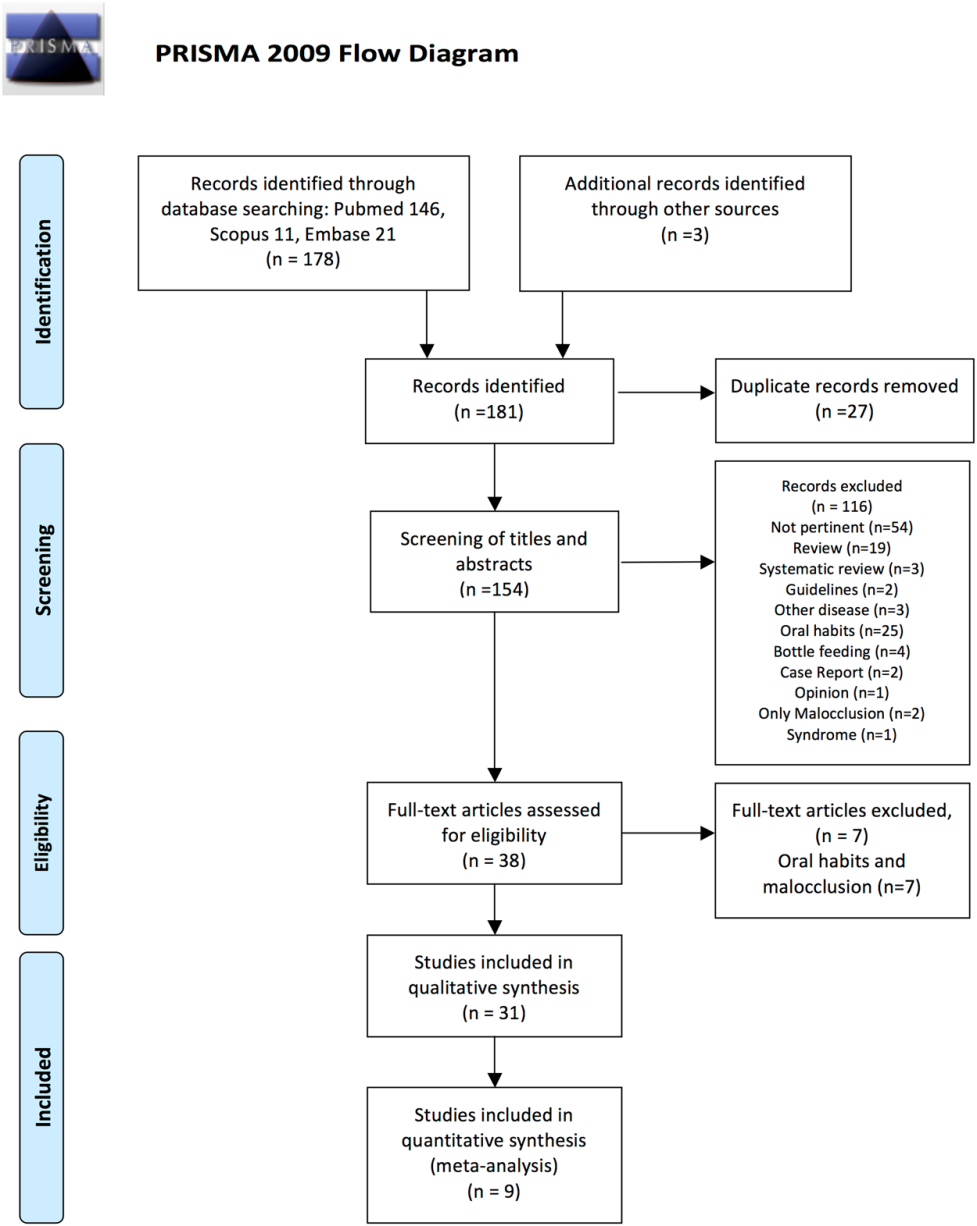
PRISMA 2009 Flow Diagram. From: Moher D, Liberati A, Tetzlaff J, Altman DG, The PRISMA Group (2009). Preferred Reporting Items for Systematic Reviews and Meta-Analyses: The PRISMA Statement. PLoS Med 6(7): e1000097. doi:10.1371/journal.pmed1000097.

The protocol registered with PROSPERO stated that the search would be limited to the year 2000 or later, but during the search it was decided not to apply a time limit in order not to exclude any article that met the other inclusion criteria. The database searches identified 178 articles (146 in Pubmed, 21 in Embase and 11 in Scopus) and hand-searching found three more. The number of duplicates removed was 27. Initial screening of the titles and abstracts of the resulting 154 articles led to excluding 116. The full texts of the remaining 38 were then read. Finally, the 31 articles that met all the eligibility criteria were included in the qualitative synthesis. The inter-examiner agreement was greater than 0.9. Nine of the studies were included in the meta-analyses (Fig. 1).

### Characteristics of the studies included

Of the thirty-one articles included in the review, twenty were cross-sectional, three longitudinal, seven cross-sectional in a study cohort, and one was a case-control study. Four examined breastfeeding and malocclusion in mixed dentition and twenty-seven in primary dentition.

The sample sizes of the studies ranged between 80 and 2026. The mixed dentition ages ranged between 6 and 15 years and the primary dentition ages between 0 and 6 years.

As regards the types of malocclusion, twelve of the studies examined the relationship between breastfeeding and posterior crossbite: Limeira *et al*. in mixed dentition^8^, and the other eleven in primary dentition^4, 9–11, 18–24^. The relationship between breastfeeding and overbite/anterior openbite was examined by fourteen studies, all in primary dentition^3, 6, 10, 12, 18–21, 24–29^. The relationship between breastfeeding and overjet/anterior crossbite in primary dentition was examined by six studies^3, 10, 18–20, 29^.

Luz *et al*. and Thomaz *et al*.^30, 31^ examined the association between breastfeeding and dental/skeletal class II in mixed dentition and Nahas-Scocate *et al*., Caramez da Silva *et al*., Feldens *et al*. and Agarwal *et al*.^13, 14, 32, 33^ investigated the same association in primary dentition. The relationship between breastfeeding and the development of occlusion/malocclusion was analyzed in nine studies, all in primary dentition^5–7, 10–12, 18, 34, 35^.

The relationship between breastfeeding and the presence/absence of interdental spaces in primary dentition was examined by three studies^9, 11, 33^. Only one study^36^ investigated the association between breastfeeding and palate depth in primary dentition, and one other^37^ looked at the association between breastfeeding and facial pattern in mixed dentition.

Tables 1 and 2 summarize the data collected from all the studies in mixed and primary dentition, respectively.

**Table 1:** Characteristics of articles studying the relationship between breastfeeding and malocclusion in mixed dentition.

Author (year) [reference] Type of study	N (dropouts) % gender (n) Mean age Sample selection Setting	Results	Odds Ratio	Quality Newcastle Ottawa Scale
Limeira *et al*.^8^ (2014) Cross-sectional	714 (-) 52.38% M (374) 47.62% F (340) 6–11 years Consecutive Brazil	Posterior crossbite greater in children not breastfed, p = 0.001	Exposure: not breastfed, Event: posterior crossbite, OR 2.25 (1.52–3.33), p < 0.001 Exposure: breastfed for less than 6 months, Event: posterior crossbite, OR 1.76 (1.09–2.84)	6
Thomaz *et al*.^31^ (2012) Cross-sectional	2026 (0) 44.1% M (892) 55.9% F (1168) 12–15 years Probabilistic stratified two-stage clusters Brazil	Breastfeeding for up to 6 months associated with Class II and Class III associated with bruxism. Short breastfeeding period associated with severely convex profile, less concave profile associated with oral breathing p < 0.05	Exposure: Breastfeeding for up to 6 months with history of nocturnal bruxism, Event: Class II, OR 3.14 (1.28–7.66) p = 0.01 Exposure: Breastfeeding for up to 6 months with history of bruxism, Event: Class III, OR 2.78 (1.21–6.36) p = 0.01	5
Sanchez Molins *et al*.^37^ (2010) Case-Control	197 (-) 53% M (105) 47% F (92) 6–11 years Consecutive Spain	Bottle fed: upper incisors protruded, dolichofacial, mandible retruded, more frequent pacifier and thumb sucking (P = 0.023) Breast fed: more brachyfacial	—	2
Luz *et al*.^30^ (2006) Cross-sectional	249 (-) -% M -% F 8.4 years Consecutive Brazil	No significant association between duration of breastfeeding and mandibular retrusion. Significant association between up to 6 months’ breastfeeding and NNSH and between NNSH and Class II malocclusion p < 0.05	Exposure: breastfeeding for up to 6 months, Event: NNSH, OR 3.81 (2.12–6.86) p = 0.00. Exposure: NNSH, Event: Class II, OR 2.4 (1.20–4.90) p = 0.02	5

M: male, F: female, OR: Odds Ratio, NNSH: non-nutritive sucking habits, (-): no information.

**Table 2:** Characteristics of articles studying the relationship between breastfeeding and malocclusion in primary dentition.

Author (year) Type of study	N (dropouts) % gender (n) Mean age Sample selection Setting	Results	Odds Ratio	Quality Newcastle Ottawa Scale
Agarwal *et al*.^23^ (2016) Cross-sectional	415 (-) 54.9% M (228) 45.1% F (187) 4–6 years Consecutive India	Exclusive breastfeeding for up to 6 months has twofold increased probability of developing nonspaced dentition	Exposure: breastfeeding for up to 6 months, Event: nonspaced dentition, OR 1.92 (1.28–2.88)	4
Germa *et al*.^24^ (2016) Prospective	422 (612) 50% M (212) 50% F (212) 3 years Consecutive France	Breastfeeding duration not associated with posterior crossbite. Anterior openbite more frequent in children breastfed for up to 6 months	—	5
Feldens *et al*.^32^ (2016) Cross-Sectional	1026 (5.4%) 52% M (534) 48% F (492) 2–5 years Consecutive Brazil	Greater distocclusion in children with shorter duration of breastfeeding	Exposure: breastfeeding for up to 6 months, Event: malocclusion, OR 1.63 (CI 1.32–2.03)	4
Lopes-Freire *et al*.^7^ (2015) Cross-Sectional	275 (-) 52.4% M (144) 47.6% F (131) 3–6 years Consecutive Spain	No significant association between breast or bottle feeding and malocclusion (p > 0.05). No association between duration of breast or bottle feeding and malocclusion.	Exposure: exclusive breastfeeding, Event: malocclusion, OR 1.37 (CI 0.34–5.51) p = 0.739	5
Peres *et al*.^10^ (2015) Prospective cohort	1123 (3108) 52.4% M (588) 47.6% F (535) 5 years Consecutive Brazil	Predominant breastfeeding (WHO) is related to less openbite, overjet and moderate-severe malocclusion (p = 0.019). Pacifier modifies associations. So does breastfeeding for overjet or posterior crossbite. Less openbite in children with 3 to 6 months’ breastfeeding (44%)	—	6
Chen *et al*.^9^ (2015) Cross-sectional	734 (113) 54.2% M (398) 45.8% F (336) 4.48 ± 0.84 years Consecutive China	Breastfeeding for up to 6 months related to more posterior crossbite (OR: 3.13) (p = 0.031) and absence of spaces in upper arch (OR 1.63). More NNSH in children with fewer months’ breastfeeding (p = 0.038)	Exposure: No breastfeeding/breastfed for up to 6 months, Event: posterior crossbite, OR 3.13 (CI 1.11–8.82) p = 0.031. Exposure: No breastfeeding/ breastfed for up to 6 months, Event: absence of spaces in upper arch OR 1.63 (CI 1.23–2.98) p = 0.038.	4
Sum *et al*.^29^ (2015) Cross-sectional	851 (24) 55.1% M (469) 44.4% F (378) 3.42 ± 1.10 years Cluster sampling China	Exclusive breastfeeding for over 6 months related to less Class II incisor relationship / overjet (p = 0.013) and greater intercanine and intermolar width (p = 0.006). No association with overbite or openbite.	Exposure: Exclusive breastfeeding for over 6 months, Event: Class II incisor relationship, OR 0.650 (0.438–0.966) p = 0.013. Exposure: Exclusive breastfeeding for up to 6 months, Event: Class II incisor relationship, OR 0.452 (0.277–0.739), p = 0.013. Exposure: Exclusive breastfeeding for over 6 months, Event: increased overjet, OR 0.511 (0.290–0.902) p = 0.021.	4
Agarwal *et al*.^23^ (2014) Retrospective cross-sectional	415 (x) 54.9% M (228) 45.1% F (187) 4–6 years Randomized India	Greater maxillary intermolar and intercanine distances when breastfed for over 6 months (p = 0.006). More posterior crossbite in children with up to 6 months’ breastfeeding (p = 0.001). Also more NNSH with up to 6 months’ breastfeeding.	Exposure: breastfeeding for up to 6 months, Event: digit sucking, OR 2.093 (1–4.37) p = 0.046. Exposure: breastfeeding for up to 6 months, Event: NNSH, OR 1.852 (0.073–9.03) p = 0.024. Exposure: breastfeeding for up to 6 months, Event: posterior crossbite, OR 7.304 (2.68–19.89) p = 0.001.	4
Moimaz *et al*.^28^ (2014) Longitudinal cohort	80 (40) -% M(-) -% F(-) 30 months Consecutive Brazil	Breastfeeding is related to overjet (p = 0.0001) and openbite (p = 0.002)	—	6
Galan-Gonzalez *et al*.^11^ (2014) Cross-sectional	298 (-) 45.3% M (135) 54.7% F (163) 3–6 years Representation of districts Spain	Better occlusion with breastfeeding than bottle feeding, more Class I canine, more diastemas and primate space, less crowding, less posterior crossbite, but not statistically significant (p > 0.005)	—	4
Correa-Faria *et al*.^12^ (2014) Cross-sectional	381 (-) 49.3% M (188) 50.7% F (193) 3–5 years Consecutive Brazil	With breastfeeding, more absence (69%) than presence (31%) of malocclusion. With no breastfeeding, more presence (54.8%) than absence (45.2%) of malocclusion (p = 0.007)	—	5
Bueno *et al*.^27^ (2013) Cross-Sectional	138 (-) -% M -% F 4–5 years Consecutive Brazil	Pacifiers were the factor most associated with openbite, overjet and posterior crossbite (p < 0.0001)	Exposure: Breastfeeding for over 6 months, Event: no overbite, OR 2.78 (1.074–7.246) p = 0.0314.	5
Caramez da Silva *et al*.^14^ (2012) Cross-sectional in a cohort	153 (80) 54.2% M (83) 45.8% F (70) 50 months ± 7.2. Consecutive Brazil	Breastfeeding (for over 12 months) protects against distocclusion (p < 0.001)	Exposure: Breastfeeding for over 12 months, Event: distocclusion, OR 0.44 (0.23–0.82)	5
Raftowicz-Wojcik *et al*.^6^ (2011) Cross-sectional	245 (2) -% M(-) -% F(-) 3–5 years Consecutive Poland	More openbite with breastfeeding for 0–6 months and over 12 months (p < 0.000). More overbite with breastfeeding for over 12 months (p < 0.01). More mesial occlusion with bottle-feeding	—	3
Nahas-Scocate *et al*.^13^ (2011) Cross-sectional	485 (-) 48.9% M (237) 51.1% F (248) 3–6 years Consecutive Brazil	With shorter breastfeeding duration, more likelihood of distal step (p < 0.001)	Exposure: No breastfeeding, Event: distal step, OR 3.54 p = 0.007. Exposure: breastfeeding for up to 3 months, Event: distal step, OR 4.10, p = 0.000	4
Romero *et al*.^26^ (2011) Cross-sectional	1377 (1323) 50.1% M (-) 49.9% F (-) 3–6 years Consecutive Brazil	Breastfed children presented less openbite (p < 0.05)	Exposure: No breastfeeding, Event: openbite OR 7.10 (p = 0.000). Exposure: Exposure: breastfed for up to 6 months, Event: openbite, OR 5.35 (p = 0.000) Exposure: breastfed for 6–12 months, Event: openbite, OR 4.30 (p = 0.000)	4
Massuia *et al*.^18^ (2011) Cross-sectional	374 (14) -%M -%F 4.2 ± 0.8 years Consecutive Brazil	Exclusive breastfeeding for up to 6 months: malocclusion more prevalent.. Exclusive breastfeeding for over six months is a protective factor against overjet and anterior openbite	—	4
Diouf *et al*.^36^ (2010) Cross-sectional	226 (-) 54.42% M (123) 45.58% F (103) 5–6 years Randomized Senegal	Combination of breast and bottle feeding causes longer and deeper maxilla than breastfeeding alone. It could not be checked whether bottle-feeding alone caused these effects (P < 0.05)	—	5
Kobayashi *et al*.^22^ (2010) Cross-sectional	1377 (-) 50.1% M (690) 49.9% F (687) 3–6 years Consecutive Brazil	More posterior crossbite in children with no breastfeeding, less in those with over 12 months’ breastfeeding (p = 0.000).	Exposure: No breastfeeding, Event: posterior crossbite, OR 4.9 (compared to 6–12 months’ breastfeeding, p = 0.0000), OR 19.9 (compared to over 12 months’ breastfeeding, p = 0.0000)	4
Leite-Cavalcanti *et al*.^35^ (2007) Cross-sectional	342 (-) 57.3% M (196) 42.7% F (146) 3–5 year Randomized Brazil	NNSH less frequent in breastfed children	Exposure: Artificial (bottle) feeding versus breastfeeding, Event: malocclusion, OR 5.34 (2.89–9.85) p < 0.001.	4
Peres *et al*.^21^ (2007a) Cross-sectional in a cohort	359 (-) 53.8% M (190) 46.2% F (169) 6 years Consecutive Brazil	With few months’ breastfeeding, greater posterior crossbite (p = 0.03)	Exposure: breastfed for up to 9 months, Event: openbite, PR 1.2 (p = 0.8–1.7) Exposure: breastfed for less than 9 months, Event: posterior crossbite, PR 7.4 (1.4–38.3)	6
Peres *et al*.^25^ (2007b) Cross-sectional in a cohort	359 (-) 53.8% M (190) 46.2% F (169) 6 years Consecutive Brazil	Breastfeeding for up to 9 months related to greater openbite	Exposure: breastfed for up to 9 months, Event: openbite, OR 2.8 (1.6–4.8) p = 0.001	5
Lescano de Ferrer *et al*.^20^ (2006) Cross-sectional in a cohort	290 (-) -% M -% F 5 years Consecutive Spain	Breastfeeding related to normal occlusion and less malocclusion. More overbite and less openbite with breastfeeding, no anterior crossbite with breastfeeding, anterior crossbite with artificial feeding. Posterior crossbite with artificial feeding, very low with breastfeeding (p = 0.06)	—	4
Lopez del Valle *et al*.^34^ (2006) Cross-sectional	540 (-) 52% F (-) 48% M (-) 28 ± 14 months Consecutive Puerto Rico	Breastfeeding associated with normal occlusion, less bottle feeding and less NNSH (p = 0.004)	—	5
Viggiano *et al*.^4^ (2004) Retrospective in a cohort	1130 (-) -% M -% F 3–5 years Consecutive Italy	Less posterior crossbite with breastfeeding, more frequent with bottle feeding (P = 0.0002)	—	5
Warren *et al*.^5^ (2002) Longitudinal	372 (328) -% M -% F 4–5 years Consecutive EEUU	No association between breastfeeding and occlusion	—	5
Karjalainen *et al*.^19^ (1999) Cross-Sectional	148 (31) 52.7% M (78) 47.3% F (70) 3 years (37.5 ± 2.2 months) Random Finland	No association between breastfeeding and openbite or overjet. Children with posterior crossbite had shorter breastfeeding period.	—	6

M: male, F: female, OR: odds ratio, CI: confidence interval, PR: prevalence ratio, NNSH: non-nutritive sucking habits, (−): no information

### Qualitative synthesis

The quality of the thirty-one observational studies was moderate to high in all cases according to the Newcastle-Ottawa Scale (Tables 3 and 4).

**Table 3:** Quality of the studies on the Newcastle-Ottawa Quality Assessment Scale for cohort studies.

Author (Year)	Selection (****)				Comparability (**)		Outcome (***)			Total Score
	1	2	3	4	5a	5b	6	7	8	
Agarwal *et al*.^23^ (2016)	*	*					*	*		4
Germa *et al*.^24^ (2016)	*	*					*	*	*	5
Feldens *et al*.^32^ (2016)	*	*					*	*		4
Lopes-Freire *et al*.^7^ (2015)	*	*	*				*	*		5
Peres *et al*.^10^ (2015)	*	*	*				*	*	*	6
Chen *et al*.^9^ (2015)	*	*					*	*		4
Sum *et al*.^29^ (2015)	*	*					*	*		4
Agarwal *et al*.^23^ (2014)	*	*					*	*		4
Limeira *et al*.^8^ (2014)	*	*		*			*	*	*	6
Moimaz *et al*.^28^ (2014)	*	*	*				*	*	*	6
Galan-Gonzalez *et al*.^11^ (2014)	*	*					*	*		4
Correa-Faria *et al*.^12^ (2014)	*	*	*				*	*		5
Bueno *et al*.^27^ (2013)	*	*	*				*	*		5
Caramez da Silva *et al*.^14^ (2012)	*	*					*	*	*	5
Thomaz *et al*.^31^ (2012)	*	*					*	*	*	5
Raftowicz-Wojcik *et al*.^6^ (2011)				*			*	*		3
Nahas-Scocate *et al*.^13^ (2011)	*	*					*	*		4
Romero *et al*.^26^ (2011)	*	*					*	*		4
Massuia *et al*.^18^ (2011)	*	*					*	*		4
Diouf *et al*.^36^ (2010)	*	*	*				*	*		5
Kobayashi *et al*.^22^ (2010)	*	*					*	*		4
Leite-Cavalcanti *et al*.^35^ (2007)	*	*					*	*		4
Peres *et al*.^21^ (2007a)	*	*	*				*	*	*	6
Peres *et al*.^25^ (2007b)	*	*					*	*	*	5
Lescano de Ferrer *et al*.^20^ (2006)	*	*					*	*		4
Luz *et al*.^30^ (2006)	*	*					*	*	*	5
Lopez del Valle *et al*.^34^ (2006)	*	*	*				*	*		5
Viggiano *et al*.^4^ (2004)	*	*	*				*	*		5
Warren *et al*.^5^ (2002)	*	*					*	*	*	5
Karjalainen *et al*.^19^ (1999)	*	*	*				*	*	*	6

Criteria: (1) Representativeness of the exposed cohort. (2) Selection of the non-exposed cohort. (3) Ascertainment of exposure. (4) Demonstration that outcome of interest was not present at start of study. (5) Comparability of cohorts on the basis of the design or analysis, (5a) for one factor and (5b) for additional factor. (6) Assessment of outcome. (7) Duration of follow-up period. (8) Adequacy of follow-up.

**Table 4:** Quality of the studies on the Newcastle-Ottawa Quality Assessment Scale for case-control studies.

Author (Year)	Selection (****)				Comparability (**)		Outcome (***)			Total Score
	1	2	3	4	5a	5b	6	7	8	
Sanchez-Molins *et al*.^37^ (2010)		*	*							2

Criteria: (1) Adequate case definition. (2) Representativeness of the cases. (3) Selection of controls. (4) Definition of controls. (5) Comparability of cases and controls on the basis of the design or analysis, (5a) for one factor and (5b) for additional factor. (6) Ascertainment of exposure. (7) Same method of ascertainment for cases and controls. (8) Non-response rate.

As regards the relationship between breastfeeding and posterior crossbite in mixed dentition, Limeira *et al*.^8^ found that no breastfeeding or a short period of breastfeeding were more often associated with this malocclusion. In primary dentition, most authors observed a greater prevalence of posterior crossbite in the no breastfeeding or breastfeeding for up to 6 months groups^4, 9–11, 19–24^. Only one study found no association between the duration of breastfeeding and posterior crossbite in primary dentition^24^.

Concerning anterior openbite, relationships have been reported between few months of breastfeeding^6, 21, 24, 25^ or absence of breastfeeding^12^ and greater prevalence of this malocclusion. Peres *et al*.^10^ and Romero *et al*.^26^ observed a greater prevalence of anterior openbite in patients with few months of breastfeeding, and Massuia *et al*.^18^ found that breastfeeding was a protective factor against anterior openbite.

In relation to overbite, the results are contradictory. Lescano de Ferrer *et al*.^20^ and Moimaz *et al*.^28^ observed greater overbite in children with over twelve months’ breastfeeding but Bueno *et al*.^27^ observed the opposite. Sum *et al*.^29^ found no association between breastfeeding and openbite or overbite.

With regard to overjet, some authors reported a relationship between longer breastfeeding and less overjet^10, 18, 29^, but Moimaz *et al*.^28^ found greater overjet in children with more than twelve months’ breastfeeding.

Lastly, Lescano de Ferrer *et al*.^20^ observed a lower prevalence of anterior crossbite in children who had been breastfed.

Some of the authors who examined the relationship between breastfeeding and dental class II found that a longer period of breastfeeding was associated with less prevalence of this class of malocclusion in mixed dentition^31^ and in primary dentition^13, 14, 32^ However, no relationship between class II and breastfeeding was found by Luz *et al*.^30^ in mixed dentition or by Karjalainen *et al*. or Agarwal *et al*.^19, 33^ in primary dentition.

Of the authors who examined the association between breastfeeding and occlusion development, three studies concluded that breastfeeding favors better occlusion^10, 11, 34^ and two found greater malocclusion prevalence in the absence of breastfeeding^12, 35^ or with up to six months’ breastfeeding^18^. In contrast, three groups of researchers found no association between these two variables^5–7^.

Three studies examined the relationship between breastfeeding and the presence or absence of interdental spaces in primary dentition. Two concluded that breastfeeding for up to six months is related to an absence of maxillary spaces^9, 33^, while the third related the existence of breastfeeding to diastema and primate spaces^11^.

Lastly, Diouf *et al*.^36^ analyzed breastfeeding and palate depth in primary dentition and found that a combination of breastfeeding and bottle feeding produced a longer and deeper maxilla than breastfeeding alone. Sanchez Molins *et al*.^37^ examined the association between breastfeeding and facial pattern in mixed dentition and found a greater prevalence of brachyfacial pattern in children who had been breastfed.

### Quantitative synthesis

Nine studies were included in the quantitative synthesis.

#### Association between no breastfeeding and posterior crossbite

**Figure 2:**
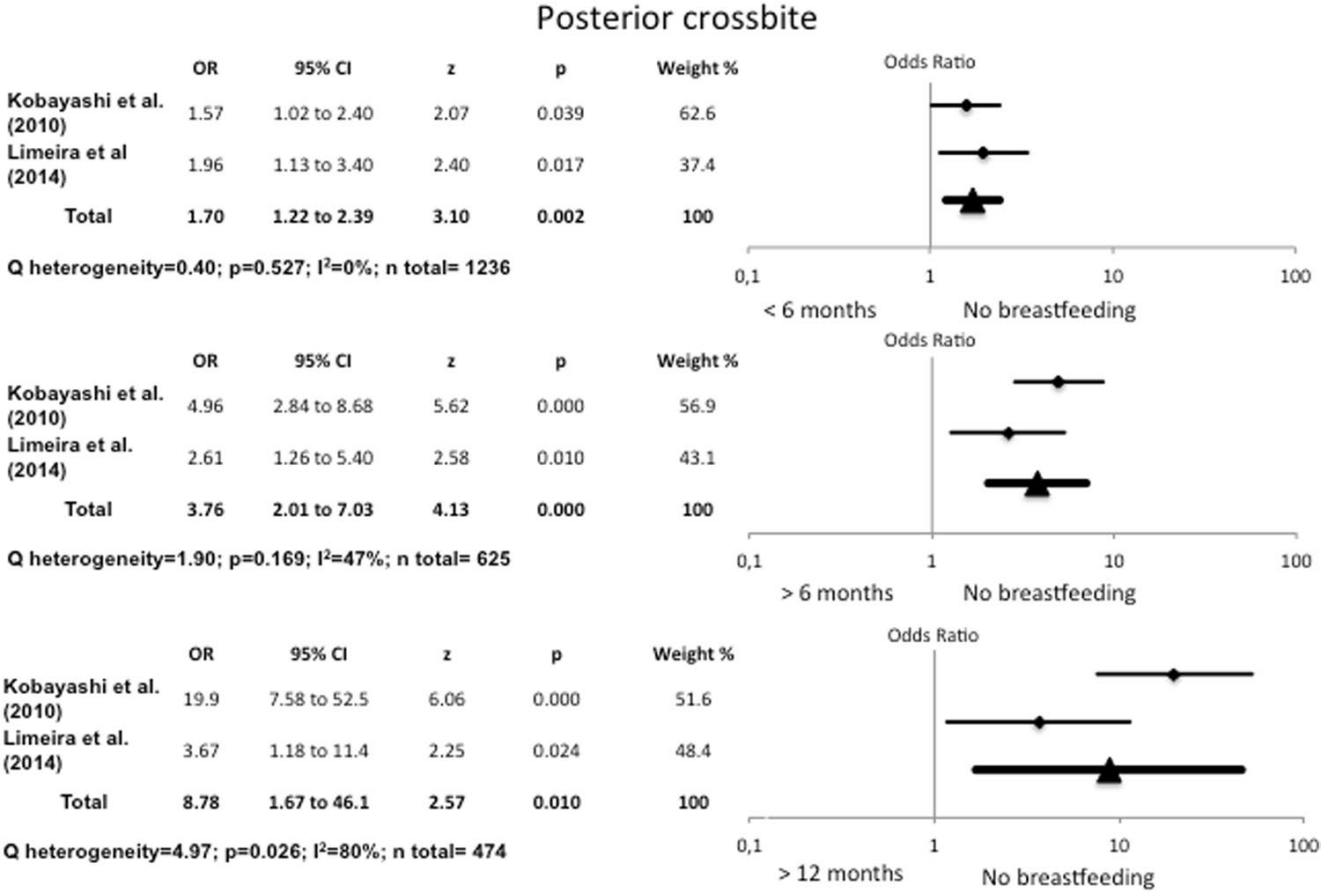
Odds Ratio for posterior crossbite, comparing no breastfeeding with breastfeeding for <6 months, >6 months and >12 months.

Children who were not breastfed presented 1.7 times more posterior crossbite than those who had been breastfed for between one and six months (OR = 1.70, 95% CI 1.22–2.39). Heterogeneity was very low (I^2^ = 0%, Q test p = 0.527). On comparing children who had not been breastfed with those who had been breastfed for over six months, the odds ratio for posterior crossbite rose to 3.76 (95% CI 2.01–7.03), with low heterogeneity (I^2^ = 47%, Q test p = 0.169). On comparing no breastfeeding with breastfeeding for over twelve months, the odds ratio rose to 8.78 (95% CI 1.67–46.1) (I^2^ = 80%, Q test, p = 0.026) **(**Fig. 2 **)**.

#### Association between exclusive breastfeeding and posterior crossbite

**Figure 3:**
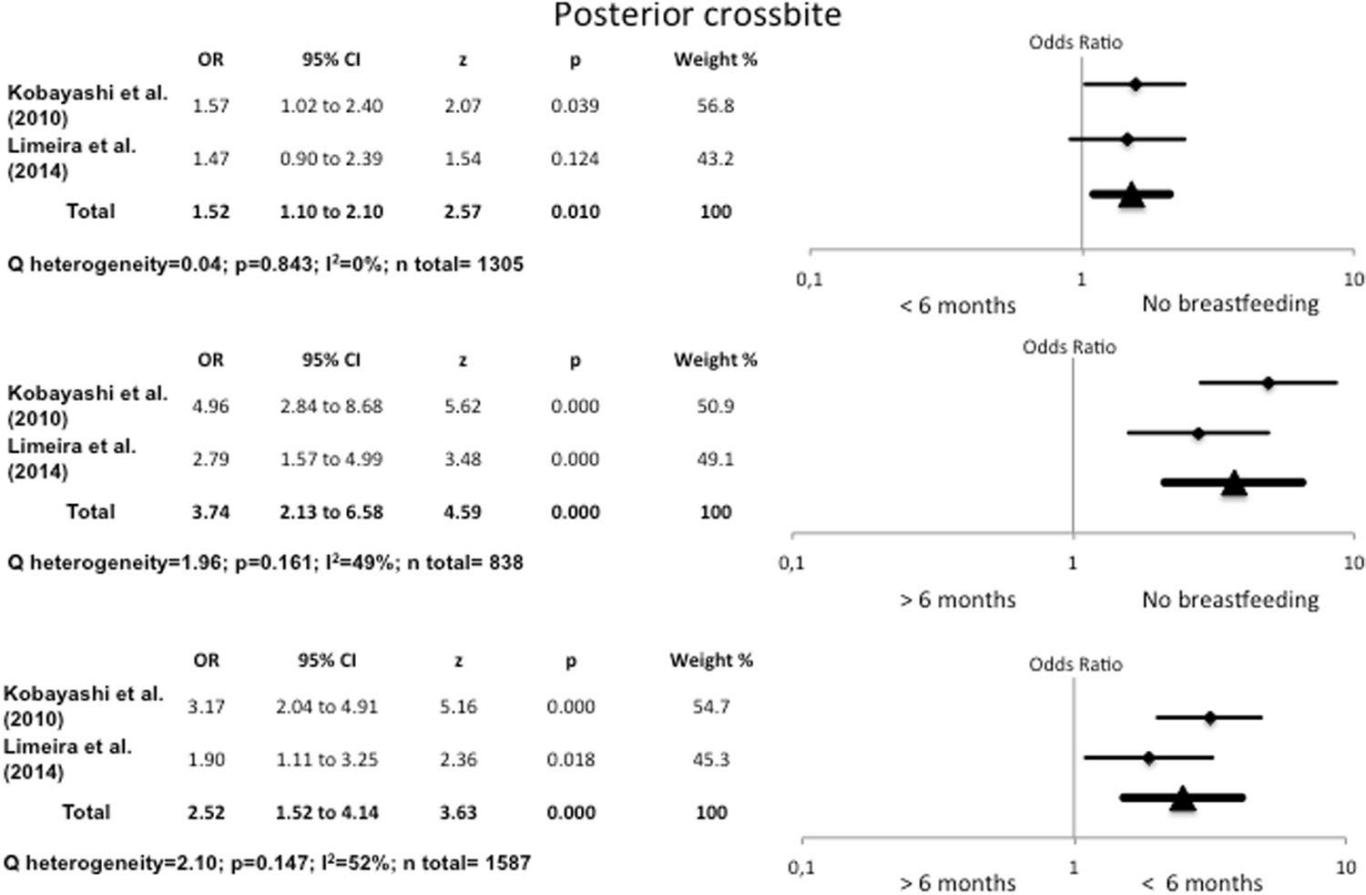
Odds Ratio for posterior crossbite, comparing no breastfeeding with exclusive breastfeeding for <6 months and >6 months, and <6 months of exclusive breastfeeding with >6 months.

The data for the exclusively breastfed groups proved very similar (Fig. 3). Children who were not breastfed presented 1.52 times more posterior crossbite than those exclusively breastfed for between one and six months (OR = 1.52, 95% CI 1.10–2.10). Moreover, on comparing children who had not been breastfed with those exclusively breastfed for over six months, the odds ratio for posterior crossbite rose to 3.74 (95% CI 2.13–6.58).

#### Association between duration of breastfeeding and posterior crossbite

**Figure 4:**
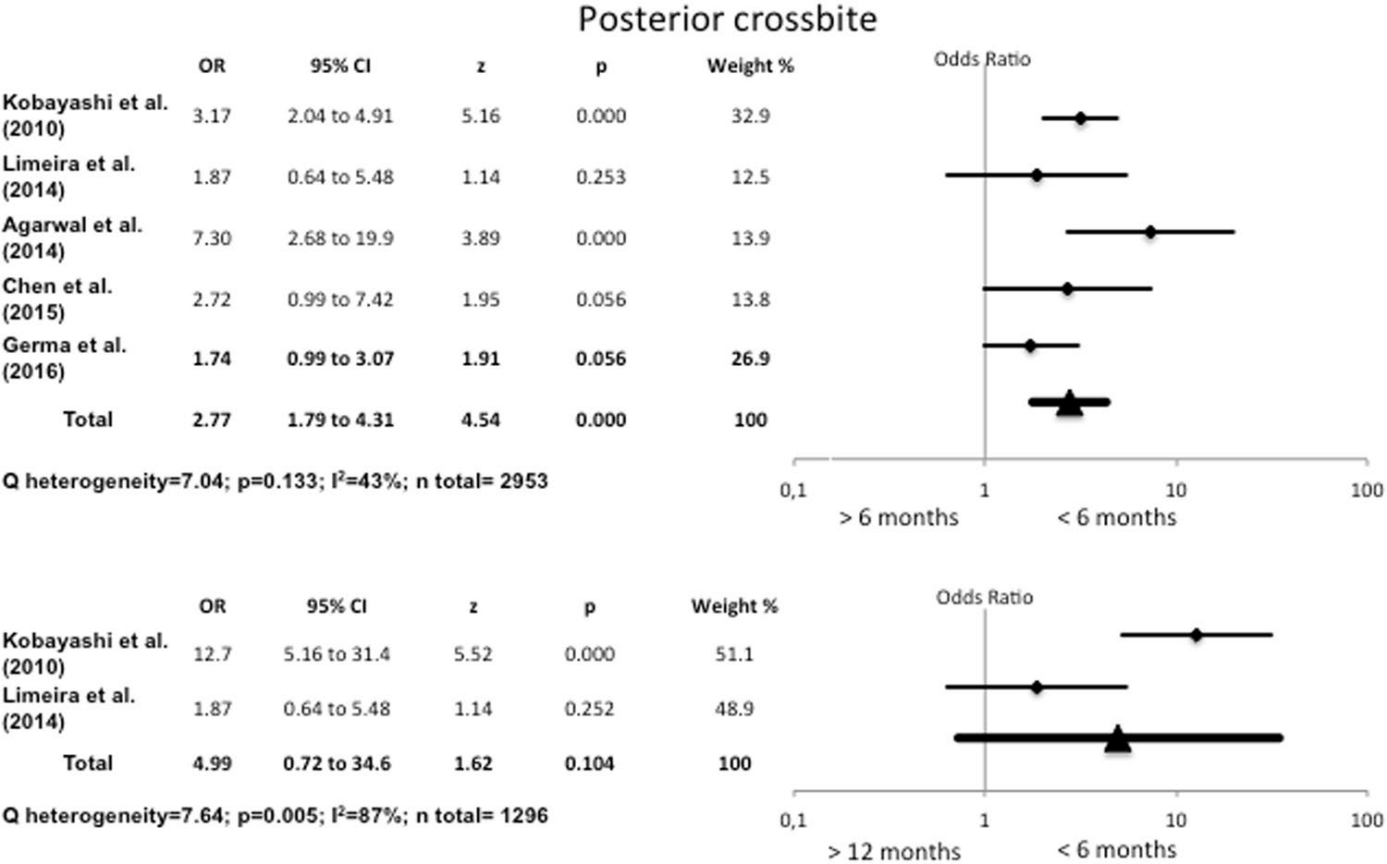
Odds Ratio for posterior crossbite, comparing breastfeeding for <6 months with breastfeeding for >6 months and >12 months.

Figure 4 shows that children who were breastfed for up to 6 months presented 2.77 times more posterior crossbite than those who had been breastfed for over 6 months (OR = 2.77, 95% CI 1.79–4.31). The heterogeneity was low (I^2^ = 43%, Q test p = 0.133). The data for exclusively breastfed children (Fig. 3) were very similar (OR = 2.52, 95% CI 1.52–4.14).

Additionally, the odds ratio in children breastfed for between one and six months was five times greater than for those breastfed for over twelve months (OR = 4.99, 95% CI 0.72–34.6), though the differences were not statistically significant. The heterogeneity was I^2^ = 87% (Q test p = 0.005) (Fig. 4).

#### Absence of association between breastfeeding duration and openbite

**Figure 5:**
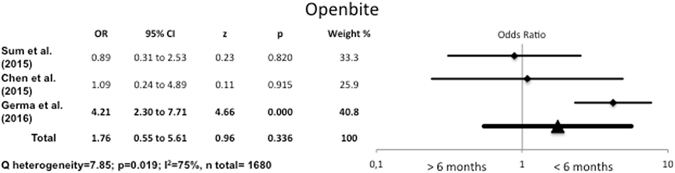
Odds Ratio for openbite, comparing breastfeeding for <6 months with breastfeeding for >6 months.

Figure 5 shows the relationship between openbite and patients who were breastfed for up to six months and for over six months. There were no significant differences between the two groups (OR = 1.76, 95% CI 0.55–5.61) and the heterogeneity was high (I^2^ = 75%, Q test p = 0.019).

#### Association between breastfeeding duration and Class II molar relationship

**Figure 6:**
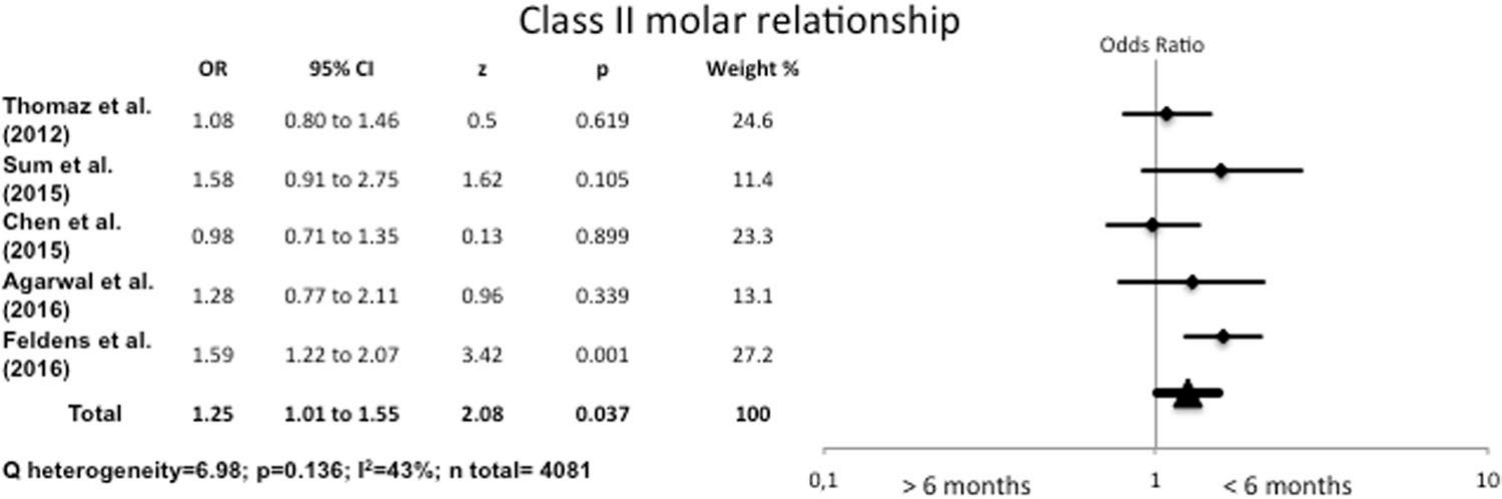
Odds Ratio for Class II molar relationship, comparing breastfeeding for <6 months with breastfeeding for >6 months.

Children who were breastfed for up to six months presented 1.25 times more Class II relationship than those who had been breastfed for over six months. There were significant differences between the two groups (OR = 1.25, 95% CI 1.01–1.55) and the heterogeneity was moderate (I^2^ = 43%, p = 0.136) (Fig. 6).

#### Association between breastfeeding duration and non-spaced dentition

**Figure 7:**
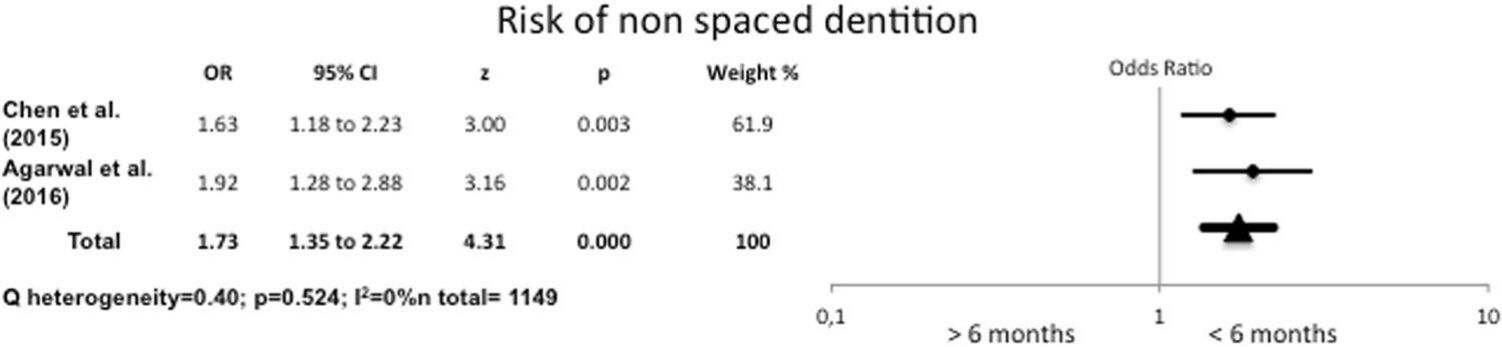
Odds Ratio for non-spaced dentition, comparing breastfeeding for <6 months with breastfeeding for >6 months.

Children who were breastfed for up to six months presented 1.73 times more non-spaced dentition than those who had been breastfed for over six months. There were significant differences between the two groups (OR = 1.73, 95% CI 1.35–2.22) and the heterogeneity was low (I^2^ = 0%, Q test p = 0.524) (Fig. 7).

## Discussion

This systematic review examines the current evidence on the possible effects of breastfeeding on occlusal development. The consensus is clear on the greater prevalence of posterior crossbite in both mixed and permanent dentition in the absence of any form of breastfeeding, and also on the association between a longer breastfeeding period and a lower prevalence of posterior crossbite^4, 8–11, 19–23^, although one study found no association^24^. A possible explanation for this association is that breastfeeding is based on advancing the mandible and raising and lowering the tongue, which promotes balanced muscular development, whereas the main action in bottle feeding is sucking, which contracts the buccinators and favors a narrower maxilla^22^.

The same consensus was not found for the other malocclusions considered. For overbite, anterior openbite and overjet, the results were disparate and no homogeneous conclusion could be reached. Regarding dental and skeletal class II malocclusion, despite some discrepancies the majority of studies considered that breastfeeding is associated with less distocclusion in both primary^13, 14, 32^ and mixed dentition^31^. However, other authors^19, 30, 33^ found no relationship between breastfeeding and class II in mixed and primary dentition, respectively.

The present meta-analysis found that the risk of posterior crossbite falls as the duration of breastfeeding rises. It also found that the risk of posterior crossbite was 1.52 times higher in children who had not been breastfed than in those exclusively breastfed for between one and six months, and 3.74 times higher than in children exclusively breastfed for over six months.

As regards openbite/overbite, the results were contradictory. While Lescano de Ferrer *et al*.^20^ and Moimaz *et al*.^28^ observed greater overbite in children with over twelve months’ breastfeeding, Bueno *et al*.^27^ encountered the opposite, and Sum *et al*.^29^ found no association. Massuia *et al*.^18^ reported that breastfeeding was a protective factor against anterior openbite. They also found that pacifiers could have a negative effect on the duration of breastfeeding, limiting its beneficial effects on occlusion development^38^.

The present meta-analysis found that children who were breastfed for up to six months presented 1.25 times more risk of Class II relationship and 1.73 times more risk of non-spaced dentition than those who had been breastfed for over six months.

A very small number of studies examined the effect of breastfeeding on palate depth and facial pattern. It would be useful for future studies to include these variables.

The sample sizes were large and representative: the smallest had 80 participants^28^ and the largest 2026^31^.

Almost all the studies collected the breastfeeding data through retrospective questionnaires or interviews with the parents, which could give rise to memory bias in the parents’ recollection of the number of months for which the children were breastfed. Only the 3 longitudinal studies^5, 10, 28^ avoided this possible bias, collecting the data prospectively.

Some limitations of the present systematic review and meta-analysis are that most of the studies did not specify whether or not breastfeeding was exclusive, or take into account possible confounders such as digit sucking or the use of a pacifier.

In an attempt to control for publication bias, the search was conducted in three databases and was complemented by a grey literature search and hand-searching.

A further limitation in this meta-analysis is a certain heterogeneity among the studies analyzed, which limited their comparability. Additionally, they were observational studies of child populations, in which it is difficult to control for confounders.

Although two systematic reviews^39, 40^ and three meta-analyses^38, 41, 42^ have been published recently, the present study provides separate assessments of the effects of breastfeeding on primary dentition and on mixed dentition, as well as a meta-analysis that examines the relations between breastfeeding and different malocclusion traits separately, including openbite, class II malocclusion and non-spaced dentition, which have not been studied in previous meta-analyses.

In conclusion, breastfeeding is a protective factor against posterior crossbite and class II malocclusion in primary and mixed dentition. The protective effect increases in line with the months of breastfeeding. There is no clear evidence for breastfeeding providing protection against other malocclusion risks such as openbite.

To avoid bias in the results would require longitudinal studies with data on the months of exclusive breastfeeding, collected prospectively through questionnaires administered to the mothers, and subsequent examination of occlusal status at the primary dentition, mixed dentition and permanent dentition stages. They should also consider confounders such as non-nutritional sucking habits and the use of baby feeding bottles.
